# Embodied mental rotation ability in open- and closed-skill sports: pilot study with a new virtual paradigm

**DOI:** 10.1007/s00221-023-06753-z

**Published:** 2024-01-20

**Authors:** Mai Geisen, Markus Raab, Petra Jansen, Stefanie Klatt

**Affiliations:** 1 Institute of Exercise Training and Sport Informatics, German Sport University Cologne, Cologne, Germany; 2 Institute of Psychology, German Sport University Cologne, Cologne, Germany; 3 Institute of Sport Science, University of Regensburg, Regensburg, Germany

**Keywords:** Sports expertise, Embodiment, Cognition, Mental representation

## Abstract

Embodied mental rotation is the influence of the body on mental rotation ability. Sports expertise enhances embodied mental rotation ability. However, sport-skill-dependent effects remain unclear. Previous studies refer to the influence of body positions on mental rotation ability. Yet, in sports, the investigation of the effect of simultaneous body and mental rotation movements is essential. Athletes need to constantly mentally and physically adapt to environmental changes and new motor tasks while being in motion themselves. This study aimed to investigate embodied mental rotation ability with simultaneous body and mental rotation in individuals with different sport skills, i.e., in open- and closed-skill sports. Forty-eight men and women, divided into two groups depending on their sport, performed 32 trials of an extended embodied mental rotation task. Simultaneous body and mental rotation were enabled by a novel test method including Virtual Reality. Results revealed shorter response times to the task stimulus in closed-skill sports participants than in open-skill sports participants. This group difference was significant for trials in which rotation directions of the own body and the mental rotation stimulus were aligned. The results might be related to sport-specific skill development processes. Motor imitation skills, as relevant in many closed-skill sports, may facilitate cognitive processes when the motion direction of the own body and of the mental rotation stimulus are aligned. The novel test method identifies potential applications that should be increasingly explored in the future, both for cognitive science and sports research.

**Supplementary Information:**

The online version contains supplementary material available at 10.1007/s00221-023-06753-z.

## Introduction

Engaging in sports activities positively impacts visual-spatial skills, such as mental rotation ability (Jansen et al. 2012; Jansen and Lehmann 2013; Pietsch and Jansen 2012; Schmidt et al. 2016; Voyer and Jansen 2017). This ability involves mentally rotating images of objects in three-dimensional space (Shepard and Metzler 1971). Yet, the effects of different types of sports on mental rotation ability may vary based on sport-specific visual-spatial demands (Pietsch et al. 2019). Mental rotation ability aligns with physical rotation ability, i.e., actually rotating objects with one's own hands. This reflects in similarities in angular disparities and response times (Gardony et al. 2014). Moreover, mental rotation ability can be influenced by the link between the bodily self and motor representations in terms of embodied cognition. Taken together, it is seen as the construct of embodied mental rotation (Kaltner et al. 2014). An individual’s body position can influence mental rotation ability (Francuz 2010). This could mean that in gymnastics, the position of parallel bars in space might be perceived differently when gymnasts are standing in front of it compared to when they are in a horizontal position on the bars. Beyond various body positions, athletes simultaneously engage in visuo-spatial cognitive processes and motor actions (Williams et al. 1999). Considering this, research should go beyond examining the effect of mere body positioning on mental rotation ability and incorporate body motion in athletes. This study used Virtual Reality (VR) technology to investigate embodied mental rotation in open- versus closed-skill sports participants during bodily rotation, addressing the need for a more comprehensive understanding of this relationship.

Open-skill sports, characterized by unpredictable and dynamic environments, involve activities influenced by external factors, like opponents and teammates. Closed-skill sports feature stable and self-paced motions, demonstrating a higher level of motor control and predictability (Heilmann et al. 2022). Athletes either experienced in open- (soccer) or closed- (gymnastics) skill sports demonstrated superior mental rotation performance compared to non-athletes (Jansen et al. 2012; Jansen and Lehmann 2013; Pietsch and Jansen 2012; Schmidt et al. 2016). Open-skills may enhance mental rotation ability through visual-spatial abilities in unpredictable environments. Closed-skills may foster mental rotation ability via precise motor control (Pietsch et al. 2019). In the context of a visual-spatial taxonomy for sports (Pietsch et al. 2018), in open-skill sports like soccer, athletes need extrinsic abilities for positioning themselves and objects in motion. In closed-skill sports like gymnastics, intrinsic abilities are crucial for maintaining balance and coordination during complex motions and rotations.

In comparison, athletes with open-skills reacted faster on mental rotation stimuli presented in front view during object-based and egocentric transformation (Pietsch et al. 2019). Similarly, participants with open-skills, i.e., in wrestling, outperformed participants with closed-skills, i.e., in running, in a mental rotation task (Moreau et al. 2012). Conversely, in a study involving open- (soccer) and closed- (gymnastics) skill sports athletes along with non-athletes, gymnasts exhibited superior abilities (Jansen and Lehmann 2013). With increasing stimulus rotation angle, male individual sports athletes in gymnastics and karate showed better mental rotation ability than male team sports athletes in volleyball and basketball (Pasand et al. 2015). Ozel et al. (2004) reported no significant impact of an individual's involvement in open- or closed-skills sports on their performance in mental rotation tasks.

While studies have examined the link between different sports and mental rotation ability, a research gap exists concerning an embodied cognition approach to mental rotation ability in individuals from diverse sports backgrounds. Embodied cognition theories propose a deep anchoring of cognitive processes in the body’s actions. Motor information available through motion experience as well as the interaction of the body with the world and its impact on cognition are focused on (Gibson 1979; Raab and Araujo 2019; Wilson 2002). In the current study, this phenomenon plays a vital role in understanding how specific motion experiences inherent to different sports may influence an individual's mental rotation ability. Kaltner et al. (2014) investigated performance differences between object-based and egocentric transformations in a mental rotation task using images of one's own or another person's body as stimuli. Results showed that human body stimuli facilitated embodied spatial transformations for egocentric tasks but not for object-based transformations. This highlights the significance of the link between the bodily self and higher cognitive processes like mental rotation, suggesting the need for further embodied mental rotation research. Gardony et al. (2014) found similar response patterns in tasks requiring mental and physical figure rotation, suggesting shared processes of mental and physical rotation. Francuz (2010) investigated how mental rotation of visual objects depends on the observer's body position and the object’s spatial orientation. The process of mental rotation was significantly altered by the interaction between body position and object orientation. This result is expressed in the fact that real objects have a natural tendency to move to a particular side as soon as the person is on the same side, indicating mental representation’s embodiment. The way we mentally perceive space is closely intertwined with our body’s evolutionary attributes (Francuz 2010).

In sports, embodied mental rotation should be investigated when one's body is not only placed in a similar position as the mental representation, but also experiences the same motion. This can be based on the common coding approach that highlights the interconnectedness of perception and action (Prinz 1997). It emphasizes a shared common representation domain of perceived events and planned actions. The visual perception of an event results in associated action. Conversely, an action results in the activation of the associated perceptual event (cf. Harris et al. 2000). Open-skill sports, like soccer, demand quick mental adjustments to align one’s motion and spatial position with the ball, other players, and the goal for accurate passes and shots. Closed-skill sports, like dancing, require performers to mentally adapt their bodies and limbs in coordination with group members to execute complex and highly precise movements (Pietsch et al. 2019). The ability to perceive, mentally adapt, and manipulate visual-spatial information in a dynamic and embodied manner is vital for success in various physical activities. Motor actions are dependent on individual perceptions (underlined by common coding approach; Prinz 1997). Given this, the goal of this study was to extend the exploration of embodied mental rotation ability, specifically of simultaneous body rotation and mental rotation, in individuals with diverse sports backgrounds. This approach aimed to enhance the understanding of the relationship between open- and closed-skill sports and cognitive skills. The study focused on the interference of an own bodily rotation and the judgment of another’s rotation. The latter can be understood as mental rotation as it relies on similar embodied elements. We refer to the possibility of the extended definition of mental rotation especially in relation to embodied cognition research (Löffler et al. 2017). Our approach is explained in more detail in the Methods section of the paper. The following research question was raised, which to the best of our knowledge has not been previously addressed: Is there a difference in embodied mental rotation ability, i.e., measured by the response time and number of errors, between humans with experience in open-skill versus closed-skill sports?

To investigate embodied mental rotation ability, we employed VR technology. Participants performed a mental rotation task using VR glasses and VR-compatible hand controllers while being physically rotated on a turntable. VR technology immerses users by visually excluding real-world elements, enabling virtual real-time information integration (Kaplan et al. 2021). High-quality head-mounted displays in the form of 3D glasses and flexible and ergonomic handling allow users to directly observe and simulate movements (Kosmalla et al. 2017; Petri et al. 2018; Sadeghi-Esfahlani et al. 2019; Taçgin 2020). Our study follows previous studies that have confirmed the use of VR as a promising tool for the development of visuo-spatial tasks, especially in relation to mental rotation tasks incorporating 3D stimuli (Lochhead et al. 2022; Tang et al. 2023).

In this study, our hypothesis is rooted in the distinction between closed- and open-skills, as suggested by Pietsch et al. (2019). We postulate that closed-skills are more associated with motor control of precise motion rotation than open-skills. Experience in closed-skill sports may facilitate mental rotation ability when the body is involved in the rotation process. In addition, closed-skill sports, like dance and gymnastics, require athletes to perform intricate and deliberate rotations as an integral part of the sport, i.e., performing a pirouette to maximize the aesthetic effect (Lott and Laws 2012; Schärli 2016). Open-skill sports, like hockey or tennis, primarily involve rotations as dynamic transitions within the overall motion sequence, rather than being the primary focus (Earp and Kraemer 2010). Based on this distinction, we propose that closed-skill sports athletes have a greater familiarity and proficiency in coordinating real body rotation with spatial processing abilities than open-skill sports athletes. This allows for more efficient and synchronized processing during embodied mental rotation. We hypothesize that closed-skill sports participants perform better in the embodied mental rotation task with shorter response times and lower number of errors across trials (including the different combinations of congruent and incongruent body and mental rotation directions), than open-skill sports participants. This hypothesis is grounded in the notion that the nature of motor experiences in different sports may contribute significantly to an individual's embodied mental rotation ability.

## Method

### Participants

Forty-eight individuals were recruited for this study. Data from 14 participants had to be excluded, because their answer accuracy within the embodied mental rotation task was not above guess probability of 50%. The present study is being based on the data from 34 participants (24.5 ± 3.64 years old, 22 women and 12 men). Data analysis of this sample size was determined based on Gardony et al. (2014) who investigated 32 participants (*n* = 16 per group) in a study on physical and mental rotation. All reported to be completely physically and mentally healthy. Seventeen participants were experienced in open-skills, i.e., basketball (*n* = 2), volleyball (*n* = 5), handball, (*n* = 3), soccer (*n* = 2), ice hockey (*n* = 1), American football (*n* = 3), and canoe polo (*n* = 1). Seventeen participants had experience in closed-skill sports, i.e., dancing [hip hop (*n* = 2), latin (*n* = 2), salsa (*n* = 2), pole (*n* = 1)], gymnastics (*n* = 5), rhythmic gymnastics (*n* = 1), acrobatics (*n* = 2), trampoline (*n* = 1), and cheerleading (*n* = 1). Table 1 provides an overview of participants' further demographic information and presents the results of statistical analyses, which indicate that there were no significant differences between participants engaged in open- and closed-skill sports in terms of their demographic characteristics. Approval was obtained from the institution’s ethics board (No. 105/2019). All participants signed a written informed consent.

**Table 1:** Overview of participants’ reports

Participants	Mean age	Duration of sports practice (in years)					Performance level				Mean training frequency (per week)	Experience in rotation practice			Experience with mental rotation tasks	
		1	2–4	5–7	8–10	> 10	Regional	National	European	Global		A lot	Little	None	Yes	No
Open-skill sports	24.6 ± 3.93	–	1	4	2	10	9	6	–	2	3.14 ± 1.12	10	7	–	3	14
Closed-skill sports	23.4 ± 3.20	1	5	4	–	7	8	4	2	3	3.12 ± 1.17	10	7	–	8	9

### Procedure and material

All participants completed a single session (approximately 30 min). A demographics questionnaire was administered and the performance level and training frequency per week of their main sport was acquired. Along Porst’s (2014) framework for creating surveys in empirical research work, response options were given on the following additional questions: the duration of practicing the sport in years, the level of experience with rotation practice, and the level of experience with mental rotation tasks. Thus, participants could indicate a tendency. Duration of sports practice in years was answered on a five-point scale with 1 = “one year”, 2 = “two to four years”, 3 = “five to seven years”, 4 = “eight to ten years”, and 5 = “more than ten years”. Rotation practice was answered on a three-point scale ranging from 1 = “a lot of experience” to 3 = “no experience”. The experience-based question on mental rotation tasks was answered on a two-point scale with 1 = “experience” and 2 = “no experience”.

**Fig. 1:**
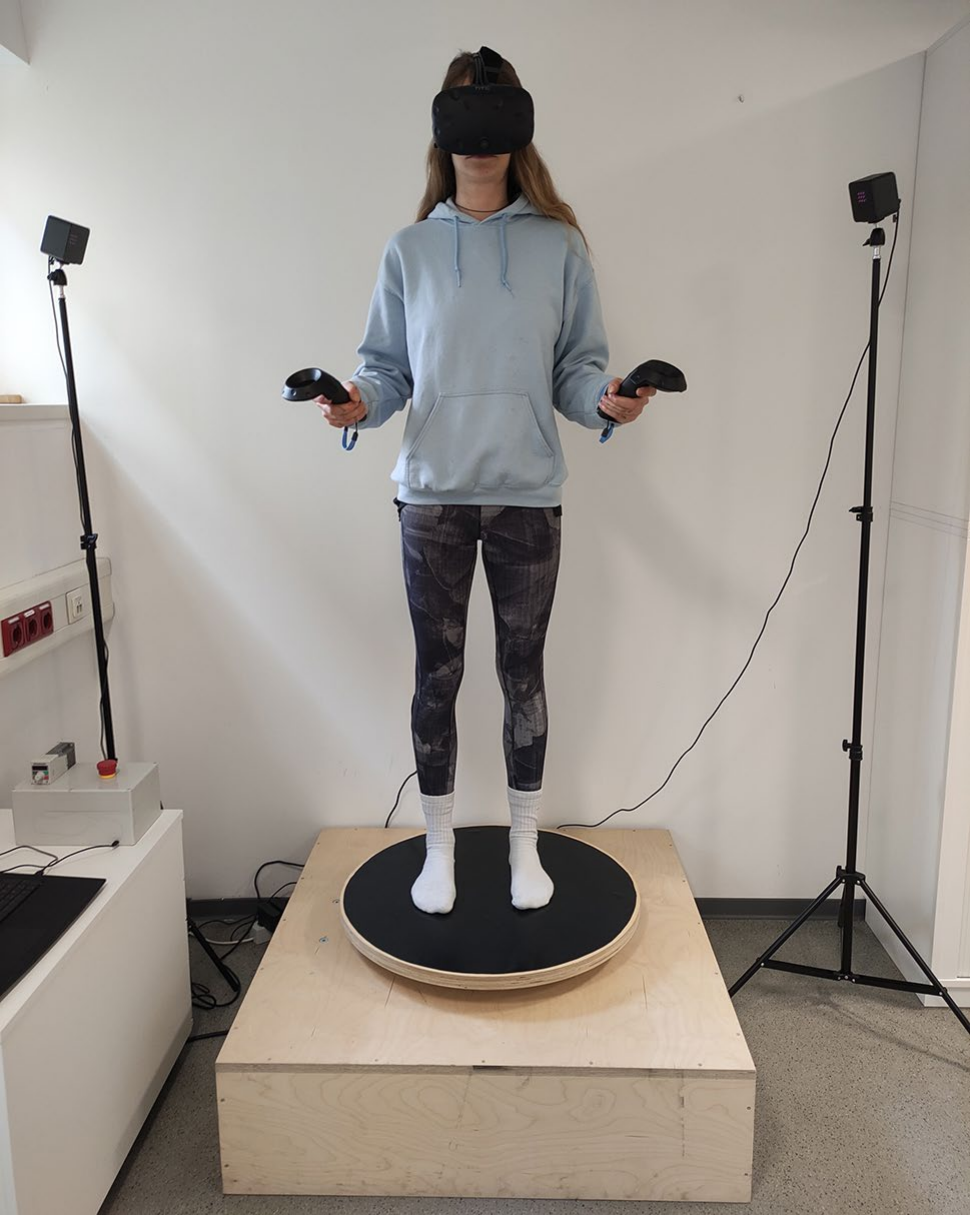
Test set-up of the embodied mental rotation task. A turntable is shown that consists of a rotating disc with a non-slip surface and a surrounding square box serving as a safety distance between the disc and the floor. While standing on the turntable, the participant is wearing VR glasses and is holding a controller in each hand. Two base stations are located on the right and on the left side of the turntable to capture the spatial position of the glasses and the controllers

As a main task, participants underwent 32 trials of an embodied mental rotation task. Meanwhile, participants stood on a turntable (cf. Fig. 1) consisting of an electrically rotating round wooden disc (70 cm ⌀) and a surrounding square wooden box (1 m^2^ and 27 cm high). The height of the wooden box was determined by the gear servo motor situated underneath the wooden construction, which was essential for controlling the rotating disc. The surface of the disc was made of a non-slip material. The box was not rotated and, in addition to covering the motor, served as a safety distance between the rotating disc and the floor. With the aid of an emergency stop switch located outside the turntable, the experimenter was able to stop the rotating disc immediately. VR glasses (HTC VIVE, https://www.vive.com) were used to display the task, while participants were passively rotating. The HTC VIVE headset offers a 360-degree experience and has a resolution of 1080 × 1200 pixels per eye. The refresh rate is 90 Hz. It is equipped with sensors, including a gyroscope, an accelerometer, and a laser position sensor. Participants were given handheld controllers, i.e., HTC VIVE controllers that enable users to interact with virtual objects. These were also programmed in Unity, so that participants could conduct the task. That is, they could respond to a stimulus shown in VR, by pressing a button on the right or left controller. Two HTC VIVE base stations, i.e., so-called "Steam VR base stations" that can track a user’s position in a space of up to 25 m^2^, were set up on tripods to the right and left of the turntable, respectively (Fig. 1).

**Fig. 2:**
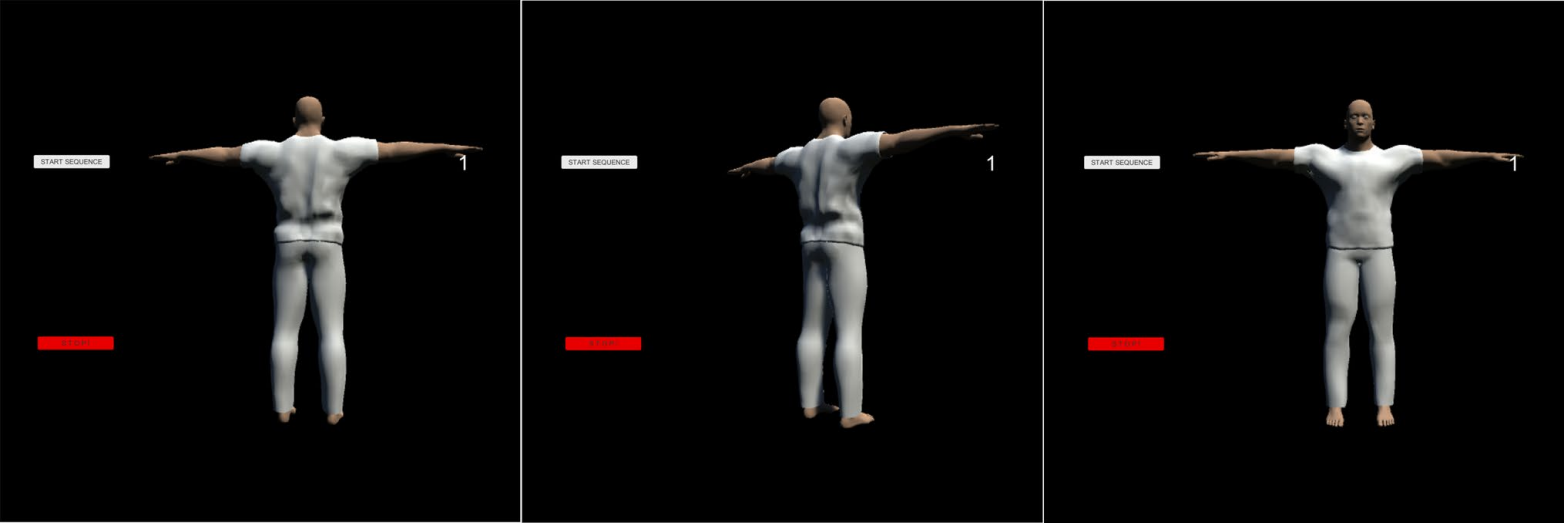
VR stimulus from various perspectives during rotation. Participants were shown a human-like figure through VR glasses. In accordance with the embodied mental rotation task, the figure was rotating and the participants had to react to it by pressing a button on a controller. The left image depicts the initial figure position, the middle image illustrates the figure's view during a rightward rotation, and the right image displays the front view of the figure after a 180-degree rotation. The buttons on the left-hand side of each image mean ‘Start sequence’ (white button), i.e., for starting the test, and ‘Stop’ (red button), i.e., for stopping the test in case of an emergency, and were operated remotely only by the experimenter via a gaming laptop. The number next to the stimulus (1) refers to the respective trial (32 trials in total) and was seen only by the experimenter

All devices, i.e., rotating disc with built-in gear servo motor and VR hardware, were controlled by a gaming specialized laptop (Medion Erazer X6807, NVIDIA GeForce GTX 1060). VR hardware was connected directly to the laptop. The rotation disc was connected to the laptop using a voltage-controlled motor unit including a microcontroller-board (Arduino). Thereby the VR stimulus could be controlled simultaneously with the rotating disc. A human-like figure was selected as a VR stimulus to enable a comparable combination of mental rotation and real rotation of the participants, i.e., creating an embodied mental rotation task. In addition, it was shown that mental rotation tasks are better performed with human-like figures than with abstract figures (Amorim et al. 2006). This was considered in the present study to avoid complicating this novel task when combining real and mental rotation. The stimulus (Fig. 2) was created in MakeHuman, an open-source 3D computer graphics middleware for the prototyping of photorealistic humanoids. It was then transferred to Unity, a game engine developed by Unity Technologies that can be used to build VR applications.

As described by Shephard and Metzler (1971), the ability of mental rotation refers to mentally representing and rotating objects in the mind. In connection with the novel research design described here, mental rotation should also be understood as the ability to mentally perceive rotations of objects. The goal was to enable simultaneous investigation of real and mental rotations under the same conditions. The implementations of the rotation conditions, both physical and mental, must therefore coincide. Accordingly, mental rotation ability can be viewed and tested as requiring the participant to react to the direction of a rotating stimulus while being passively rotated. The construct mental rotation is usually derived from classical tests but can also be adapted and implemented in a differentiated manner, taking into account extended definitions, especially in relation to embodied cognition research (Löffler et al. 2017).

Based on piloting, both the rotating disc and the VR stimulus rotated with an acceleration phase of 1 s and an extended stable speed of 5.3 revolutions per minute. They were programmed to start rotating at the same time. The speed was kept relatively slow for safety reasons. Thus, participants would not be in danger of falling off the rotating disc. Also, it should prevent participants from suffering under dizziness. The duration of acceleration was chosen on the one hand because of technical requirements. In that way, the rotating disc could reach the chosen maximum speed for the experiment. On the other hand, the participants’ perception of real rotation was to be ensured. Rotational motion perception decays at a constant velocity, especially when moving in darkness (Nooij et al. 2016). Indeed, in the present study's VR environment, participants were completely shielded from the outside world. They saw only the stimulus surrounded by a dark environment. Through the piloting process, we also confirmed that participants were adequately capable of performing the subsequent embodied mental rotation task.

### Embodied mental rotation task

Participants were divided into two groups that differed in terms of their primary sport (open-skill and closed-skill sports). Both groups performed the same task. Participants were given verbal instructions on the visualization in VR and the task. They were told that the rotating disc would passively rotate them. However, they were unfamiliar with the sequence of rotation directions of both the disc and the VR stimulus. When entering the turntable, participants were provided with the VR glasses and the controller. The experimenter started the task by pressing the ‘Start sequence’-button via the gaming laptop. Participants were then passively rotated by the disc either to the right or to the left. Simultaneously, they saw the VR stimulus also rotating to the right or to the left. The participants’ task was to decide as quickly as possible in which direction the stimulus was rotating. Meanwhile, they were being rotated themselves. For making their decision, they had to press the index finger button on one of the controllers, i.e., on the right controller for deciding that the stimulus was rotating to the right and on the left controller for deciding that the stimulus was rotating to the left. As soon as participants had pressed the button, both the disc and the stimulus stopped rotating. This was done to give participants a short break between trials in which they could perceive that both the disc and the stimulus were not moving anymore. The following trial started after 2 s. The combinations of simultaneous rotation of the disc (physical rotation) and the VR stimulus (mental rotation) were programmed as follows: Half of the task consisted of trials with congruent rotation directions of the disc and the stimulus, i.e., both to the right (*n* = 8) as well as both to the left (*n* = 8). The other half of the task consisted of trials with incongruent rotation directions, i.e., disc rotation to the right and stimulus rotation to the left (*n* = 8) as well as disc rotation to the left and stimulus rotation to the right (*n* = 8). The total number of 32 trials comprised a pre-programmed random order of 16 trials. This was repeated once. The same sequence of trials was used for all participants. By the number of trials, possible accidental errors when responding via the controller could be considered. It was decided against an even higher number of trials considering the risk of dizziness that can occur with orientation disturbances. Additionally, piloting confirmed that participants did not receive any practice with this number and order of trials, thereby ruling out order effects. This means that the impulses which sensory orientation systems are sending to the brain do not match, such as the rotating sensation of the vestibular organ in contrast to visual information in VR (Limmroth 2002).

### Data analysis

Decision responses given by the controllers were used to determine the participants' performance in the embodied mental rotation task. After completing the task, all responses of a participant were automatically sent to the gaming laptop and stored as a CSV file. The response time (sec.), described as *RT* in the following, and the number of errors (average value with decimals), described as *NE* in the following, on rotation combinations represent the values of task performance investigated in this study. Further, *RT* on congruent rotation combinations (*RTcon*) and on incongruent rotation combinations (described as *RTincon* in the following) as well as *NE* on congruent rotation combinations (described as *NEcon* in the following) and on incongruent rotation combinations (*NEincon*) were considered separately. Each value was determined by averaging the responses of a participant, i.e., averaging responses on all trials (resulting in *RT* and *NE*), averaging all responses on trials with congruent rotation directions (resulting in *RTcon* and *NEcon*) and averaging all responses on trials with incongruent rotation directions (resulting in *RTincon* and *NEincon*).

Statistical analyses have been carried out using IBM SPSS Statistics 29. Participants' task performances were analyzed, including at different rotation combinations. We determined if there were any differences in performance between open-skill and closed-skill sports participants. To measure task performance in terms of *RT, RTcon,* and *RTincon* as well as *NE, NEcon,* and *NEincon*, we performed an independent samples t test for each value. The differences between groups’, i.e., open-skill and closed-skill sports participants, with respect to the mentioned values were calculated. For the graphical presentation of the normal distribution of *RT* and *NE*, refer to the supplementary material. Effect sizes were assessed using Cohen’s d and interpreted in line with Cohen (1988). Thus, a small-effect size is found at *d* = 0.2, a medium-effect size is found at *d* = 0.5, and a large-effect size is found at *d* = 0.8.

As this study did not attain the requisite sample size to achieve a power of 0.80, a post hoc power analysis was conducted that revealed that the statistical analysis maintains a moderate level of power at 0.68.

## Results

**Fig. 3:**
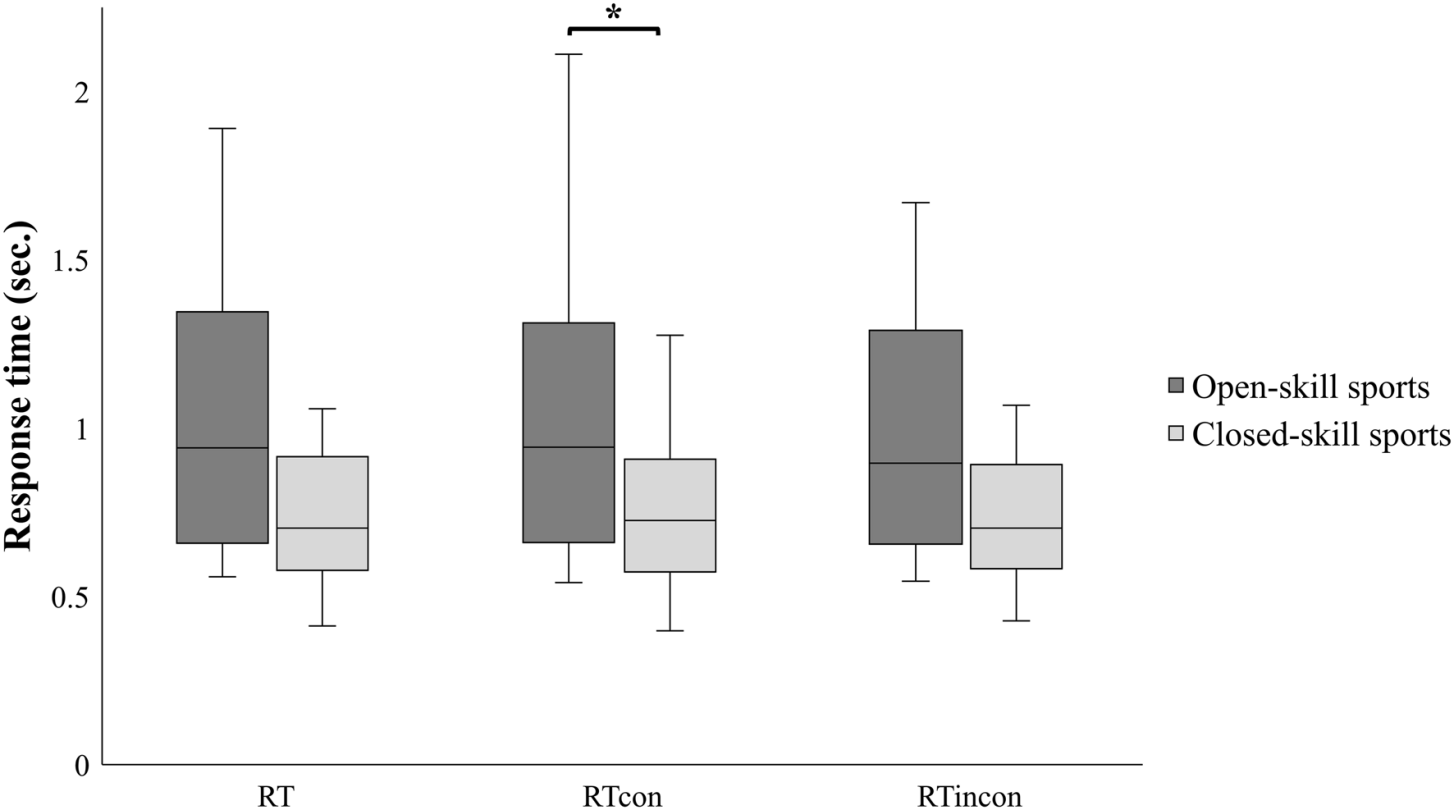
Response times on all trials (*RT*) and on congruent (*RTcon*) and incongruent (*RTincon*) rotation combinations. The embodied mental rotation task performance in terms of response times on all trials as well as on congruent and incongruent rotation combinations of the rotating disc and the VR stimulus are shown separately for each group, i.e., participants with open-skill sports experience and participants with closed-skill sports experience. * Indicates a significant difference (*p* < 0.5) between groups

Do participants with closed-skills perform better in an embodied mental rotation task than participants with open-skills? Our findings suggest that they do. Results showed a trend for a higher *RT* in open-skill sports participants (1.00 ± 0.40 s., i.e., 31.80° ± 12.72° rotation of both the rotating disc and the VR stimulus, hereinafter only referred to as °) compared to closed-skill sports participants (0.78 ± 0.27 s., i.e., 24.81° ± 8.59°), *t*(32) = 1.90, *p* = 0.067, *d* = 0.65. Significant differences were found for *RTcon* between open-skill sports participants (1.03 ± 0.44 s., i.e., 32.76° ± 14.00°) and closed-skill sports participants (0.77 ± 0.22 s., i.e., 24.49° ± 7.00°), *t*(23.50) = 2.17, *p* = 0.041, *d* = 0.74 (Fig. 3). No significant difference was found for *RTincon* between open-skill sports participants (0.97 ± 0.36 s., i.e., 30.85° ± 11.45°) and closed-skill sports participants (0.79 ± 0.33 s., i.e., 25.12° ± 10.49°), *t*(32) = 1.53, *p* = 0.135, *d* = 0.53.

**Fig. 4:**
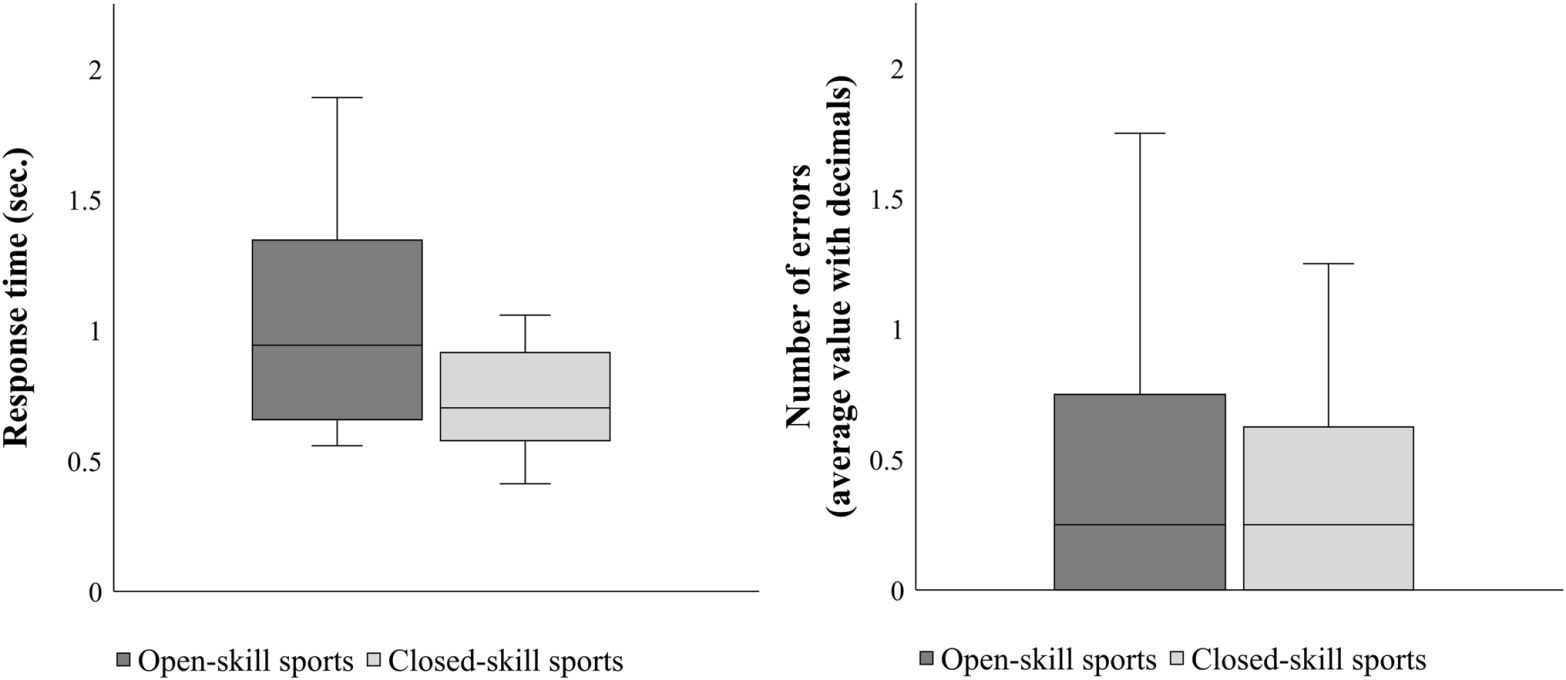
Comparison of response times on all trials (*RT*) and number of errors on all trials (*NE*). The embodied mental rotation task performance in terms of response times on all trials as well as on number of errors on all trials are shown separately for each group, i.e., participants with open-skill sports experience and participants with closed-skill sports experience

There were no significant differences for *NE* between open-skill sports participants (0.34 ± 0.40) and closed-skill sports participants (0.59 ± 0.89),* t*(32) = 1.052, *p* = 0.301, *d* = -0.361. Results yielded no significant differences in *NEcon* between open-skill sports participants (0.38 ± 0.55) and closed-skill sports participants (0.59 ± 0.92), *t*(32) = 0.792, *p* = 0.434, *d* = -0.272. Likewise, no significant differences were found for *NEincon* between open-skill sports participants (0.29 ± 0.37) and closed-skill sports participants (0.59 ± 0.97), *t*(32) = 1.171, *p* = 0.250, *d* = -0.402. As a summary of the overall results, Fig. 4 presents a comparison of both differences for *RT* and *NE* between open- and closed-skill sports participants.

Participants, whose test data were excluded from the analyses because their answer accuracy was not above guess probability of 50% (the average error rate of all other participants was 2%), had an error rate of 100% (*n* = 1), 97% (*n* = 6), 94% (*n* = 4), 88% (*n* = 1), 59% (*n* = 1), and 53% (*n* = 1).

## Discussion

In the present study, we investigated embodied mental rotation ability in individuals with open-skills versus closed-skills. Results revealed a trend for faster responses on the task in closed-skill sports participants compared to open-skill sports participants. This group difference was found to be significant for task trials in which both the own body and the mental rotation stimulus were rotating in the same directions. That is, they were either both rotating to the right or both rotating to the left. Group differences were non-significant for trials in which body and mental rotation occurred in incongruent directions. In our study, the sample size for data analysis was determined based on the work of Gardony et al. (2014). With respect to our post hoc power analysis, we contend that the discerned significant effect likely reflects inherent disparities in cognitive processing between individuals engaged in open- and closed-skill sports in mental rotation tasks. Nevertheless, it is imperative to acknowledge the need for future investigations aimed at replicating these findings with a higher statistical power.

The results of the present study are partially consistent with our hypothesis that closed-skill sports participants would react faster on the task than open-skill sports participants. The heightened sense of motor control in closed-skill sports participants (Heilmann et al. 2022) and the fact that intricate and deliberate rotational movements are often performed with a primary focus in closed-skill sports (Lott and Laws 2012; Schärli 2016) may have contributed to a facilitated spatial processing of stimulus rotation (mental rotation ability) in coordination with real body rotation. This resulted in enhanced embodied mental rotation ability. A significant performance difference between closed-skill and open-skill sports participants was found at congruent rotation directions but not at incongruent rotation directions. It suggests that both open- and closed-skill sports participants possessed similar capabilities to perform the task accurately, with the key difference being the speed of their responses (cf. Figure 4). This could be referred to the following: Individuals experienced in closed-skill sports such as dance (*n* = 8, closed-skill sports participants were experienced in sports that contain dance skills) typically acquire and refine their skills through extensive practice by imitating their coaches’ movements (Laland et al. 2016). This unique training approach may contribute to their ability to quickly react to movements by imitating them. In contrast, in open-skill sports such as soccer, skill development occurs rather through the integration of multifaceted components. This refers to technical, tactical, physical, and psychological components. Effective execution of movements at opportune moments is a primary focus (Duncan et al. 2022). As a result, dance athletes may have an advantage in quickly reacting when the mental rotation stimulus matches their own body's motion direction, while they rotate themselves. The shared rotational movement may tap into their experience of imitating movements. This in turn facilitates faster responses. Overall, these possible explanations highlight the role of skill development in shaping the differences observed in embodied mental rotation performance between closed-skill and open-skill sports participants. Based on embodied cognition perspectives (Raab & Araújo 2019), particularly the relationship between perceptual processes and motion actions (common coding approach by Prinz 1997), and different sport-specific visuo-spatial demands (Pietsch et al. 2019), the type of sport experience seems to play a role in embodied mental rotation ability.

Following new insights into neural processes underlying continuous action monitoring (e.g., Wilken et al. 2023), the results of our study may provide further insight into possible neural relationships between sensorimotor practice and motor cognition. Previous research has shown that the primary motor cortex (M1) exhibits activation patterns during time windows related to body-related mental rotation tasks (Perruchoud et al. 2018). Open-skill sports heavily rely on sensorimotor integration, as athletes must coordinate their motions based on abundant external sensory input related to their teammates, opponents, and the surrounding environment. Closed-skill sports may prioritize internally driven motor control, with athletes primarily integrating sensory input related to their own body's motions (Heilmann et al. 2022). In the context of the self-centered mental rotation task employed in our study, which engages the neural activity in M1, it is reasonable to assume that neural mechanisms favoring M1 activation are inherently more developed in athletes with closed-skills than in athletes with open-skills. This neural perspective could offer a plausible explanation for why closed-skill sport athletes exhibited faster response times. Nevertheless, we did not perform any neuronal measurements in our study, so we can only make tentative assumptions. Further research is needed to explore our findings in more detail and elucidate the underlying mechanisms driving the observed differences.

With respect to the number of errors in task performance, our hypothesis does not match with the results that no significant differences were found between groups. On average, error rates for both groups were less than 1, i.e., 0.34 ± 0.40 errors in open-skill sports participants and 0.59 ± 0.89 errors in closed-skill sports participants. A ceiling effect could be present, assuming that the task had a very low level of difficulty. Thus, group differences were only noticeable in the response times of participants. That is, open-skill sports participants took slightly longer for their decision than closed-skill sports participants. Regardless of the simplicity of the task, the fact that open-skill sports participants possess distinctive decision-making abilities (Duncan et al. 2022) may have also contributed to the results. Closed-skill sports participants, on the one hand, might have an advantage in reacting to congruent body and mental rotation directions. This might be due to their experience of imitating movements (Laland et al. 2016). Open-skill sports participants, on the other hand, might have particular abilities to make correct decisions. These are especially noticeable in the results of the number of errors. Further investigations with different levels in task difficulty should be conducted to verify this assumption.

Our results align with certain previous studies on mental rotation (Jansen and Lehmann 2013; Pasand et al. 2015). However, they conflict with other prior research findings, which indicated that open-skill athletes outperformed closed-skill athletes in mental rotation tasks (Pietsch et al. 2019; Moreau et al. 2012), or that no differences between both types of sports existed (Ozel et al. 2004). These discrepancies may arise from variations in study methods and task designs. Notably, our study primarily focuses on an embodied mental rotation task, representing a novel study method distinct from study methods on traditional mental rotation tasks, i.e., our method involved standing on an electric turntable while viewing a mental rotation stimulus through VR glasses. This shift in perspective might account for the differences in our findings compared to the previous studies that investigated mental rotation without considering embodied aspects. Moreover, our study explored a distinct dimension of mental rotation, i.e., mental rotation as the ability to mentally perceive rotations of objects. This passive mental rotation differs from the majority of mental rotation studies, which employ the Mental Rotation Test (Vandenberg and Kruse 1978) or modified versions of it (Alexander and Evardone 2008; Peters et al. 1995). The test requires participants to actively mentally rotate a set of figures around the left/right as well as the top/bottom axis. The deviation of our study design from this can be considered a limitation of our study. Nevertheless, while our study design and the results may differ from those in some earlier studies, they contribute to a broader understanding of the relationship between sports expertise and cognitive abilities within the context of embodied mental rotation.

Participants with conspicuously high error rates in task performance were both individuals with open-skills (*n* = 8) and individuals with closed-skills (*n* = 6). Thus, the results cannot be attributed to the respective sport skill. Based on descriptive statistics, no other characteristics of these participants who differed from the other participants were found either. Technical errors of the test devices can be ruled out, as an experimenter monitored the data collection throughout the entire time. The number of wrong decisions made by these participants corresponded similarly to the number of correct decisions of the participants included for data analysis. Assumingly, from the perspective of these 14 participants, they mostly decided correctly when reacting to the mental rotation stimulus. According to previous findings, perception and attention of the visual world can differ across individuals. Visual attention differences in individuals during dynamic scene viewing were associated with differences in eye movement pattern, i.e., number of fixations, fixation durations, saccade amplitudes, and fixation distances from the center of a visual scene (Holm et al. 2021). Davidoff (2015) summarized that the identical external stimulus does not inevitably elicit an identical perception in each individual. Previous research has not reported any comparable findings on differences in mental rotation perception. Thus, the phenomenon found in our study might be related to the additional embodied cognition processes during the mental rotation task. In this study, a total of 14 out of 48 participants were found to produce these conspicuously high error rates. Future research should, therefore, consider this phenomenon in relation to investigations on rotation perception, mental rotation, and embodied cognition. Gaze registration should be added to future investigations on our embodied mental rotation task.

It should be noted that our participants sample primarily consisted of individuals involved in game sports (e.g., basketball) as representatives of open-skill sports and compositional sports (e.g., dancing) as representatives of closed-skill sports. Hence, sports such as individual combat sports were not considered. On the one hand, future studies should broaden the participants sample by including a wider variety of sports. A more comprehensive exploration of the connection between embodied mental rotation and open- versus closed-skill sports would be enabled. Also, exploring more sports could shed light on varied rotation perceptions, given the high error rates in some participants in our study. On the other hand, we acknowledge that within the categories of open- and closed-skill sports, there can be differences in the cognitive demands placed on athletes. To refine group comparisons and enhance precision in further studies, researchers may also opt to select participants from more homogeneous subgroups within open-skill and closed-skill sports. Furthermore, even though we conducted pilot tests to minimize potential discomfort in VR environments as well as during passive rotation on the electric turntable, a dedicated questionnaire to assess dizziness or cyber sickness was not included. Forthcoming investigations should consider incorporating standardized assessments. In further research approaches, evaluating visually induced motion sickness or cyber sickness could provide valuable insights into whether these phenomena correlate with or affect the output measures of embodied mental rotation.

In summary, the present study used VR to extend the research on embodied mental rotation in relation to performance differences between open-skill and closed-skill sports participants. Building upon our research findings, further aspects should be considered in future investigations: In our study, embodied mental rotation ability was examined in connection with passive body rotation. This enabled the initial investigation of embodied mental rotation with physical and mental rotation. Further, in conjunction with VR technology, it allowed for standardized testing. Nevertheless, the development of a test design to examine active body rotation combined with mental rotation would be the next step forward. Thereby, even more practical statements could be made about embodied mental rotation ability. Additionally, in the present study, a single mental rotation stimulus (male virtual figure) was used. Future research should refer to further mental rotation stimuli (i.e., female figure) used in the previous studies (Città et al. 2019; Pietsch et al. 2019). These could be displayed while actually rotating.

## Conclusion

Our study investigated sport-skill-dependent differences in embodied mental rotation ability. The focus was on enabling simultaneous body rotation and mental rotation. Therefore, VR technology and an electric turntable were used. Individuals with closed-skills were found to react faster on the mental rotation stimulus shown in VR when compared to individuals with open-skills. This difference was particularly found in task trials in which rotation directions of the own body and the mental rotation stimulus were aligned. The results might be related to different skill development emphases in open-skill and closed-skill sports. In particular, skills in imitating movements, as it is relevant in many closed-skill sports, may facilitate cognitive processes when the movement of the own body and the mental rotation stimulus are aligned. Extended research is needed to make specific practical statements about embodied mental rotation abilities in sports. Moreover, novel test methods, such as those developed for our study, provide multiple potential applications. For instance, it could be used for cognitive performance diagnostics in competitive sports or to practice coordination skills. Practical application options should be increasingly explored in the future, both in cognitive and sports science.

### Supplementary Information

Below is the link to the electronic supplementary material.

Supplementary file1 (TIF 285 KB)

Supplementary file2 (TIF 244 KB)

Supplementary file3 (DOCX 90 KB)

## Data Availability

The datasets generated during and/or analyzed during the current study are available from the corresponding author on reasonable request.
