# Supplementary figures and images for: Embodied mental rotation ability in open- and closed-skill sports: pilot study with a new virtual paradigm
Source: Exp Brain Res. 2024 Jan 20;242(3):653–64. doi: 10.1007/s00221-023-06753-z (PMC10894766; doi:10.1007/s00221-023-06753-z)

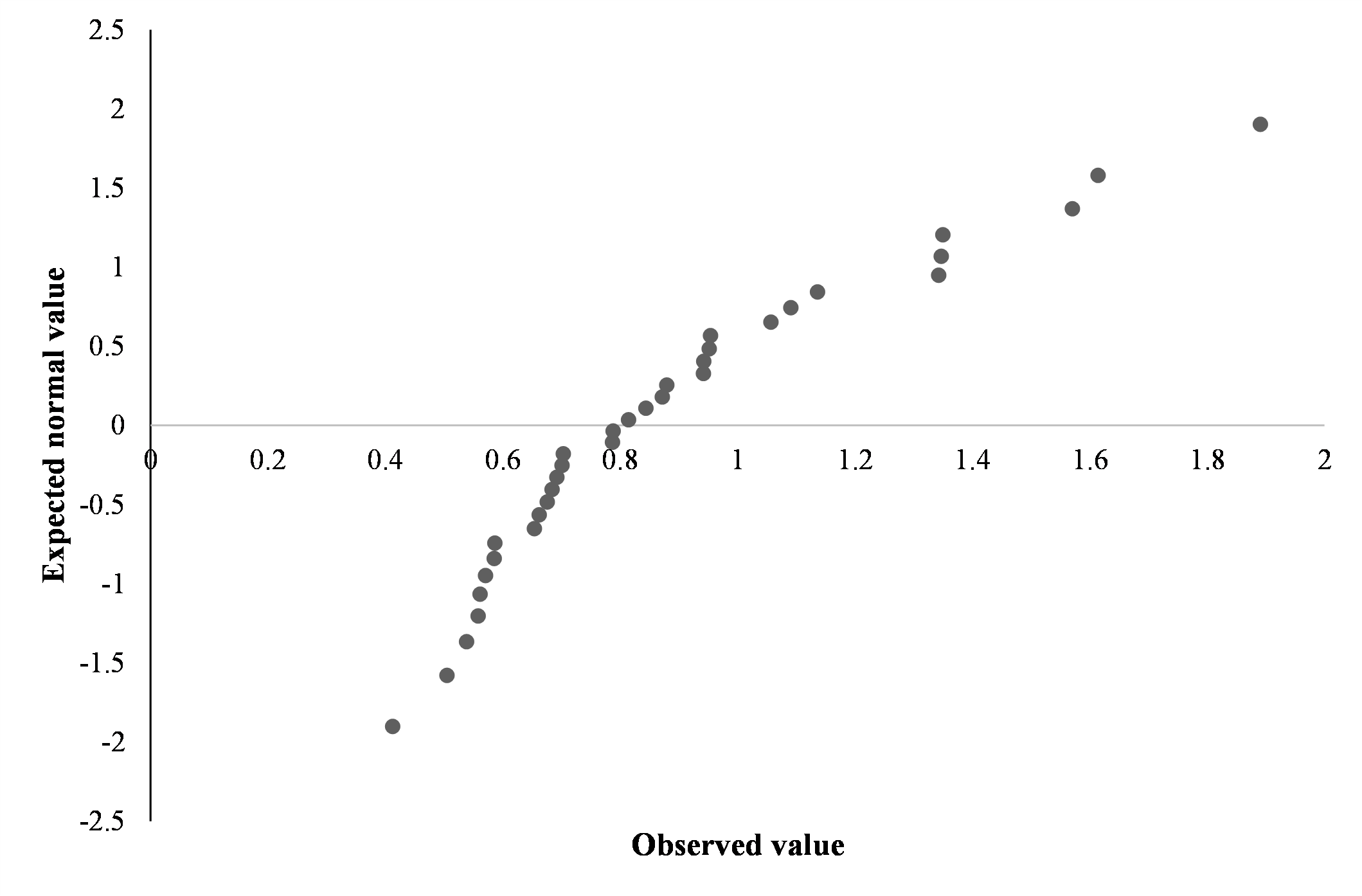

Supplement: Supplementary file 1 — Supplementary file1 (TIF 285 KB) [file 221_2023_6753_MOESM1_ESM.tif]

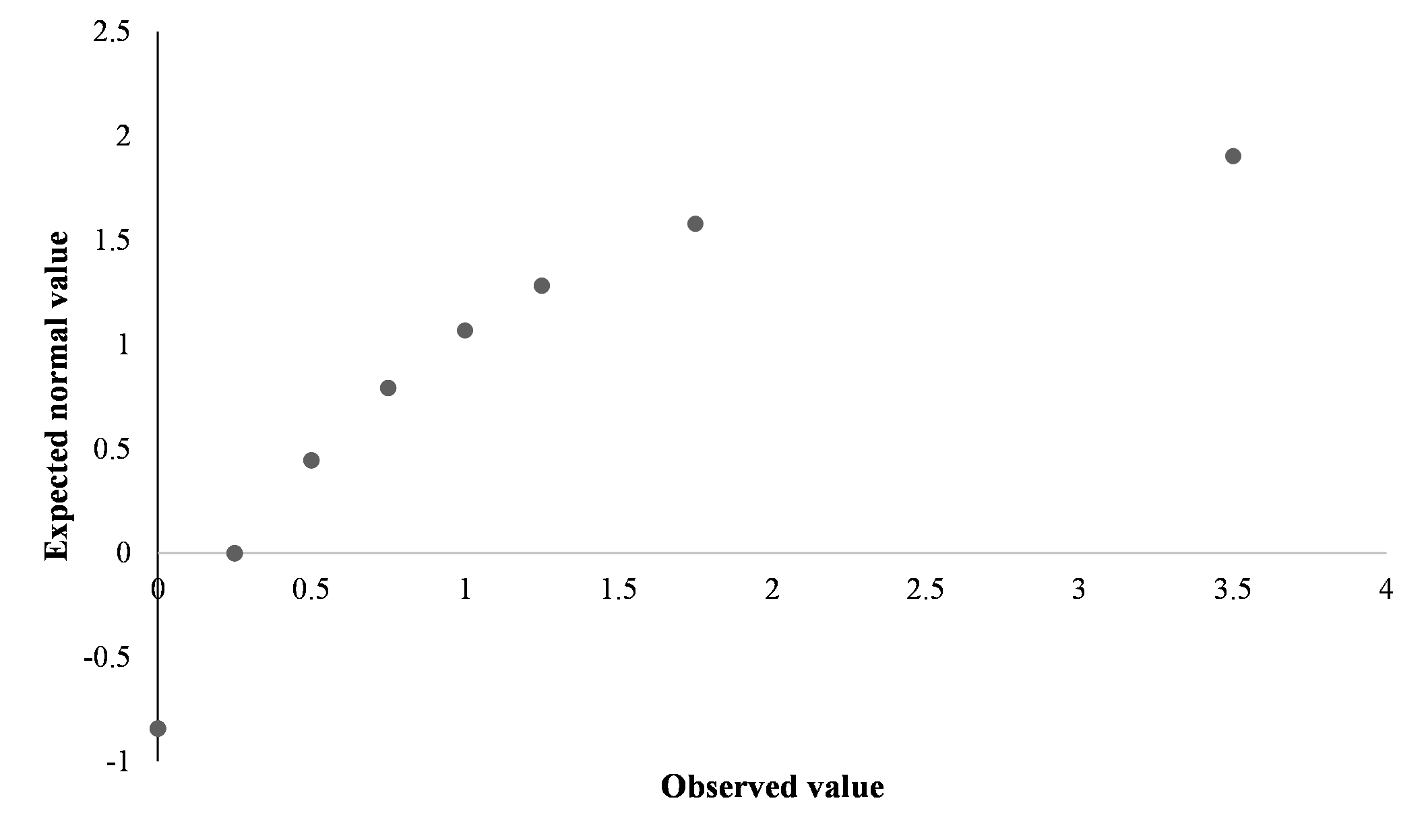

Supplement: Supplementary file 2 — Supplementary file2 (TIF 244 KB) [file 221_2023_6753_MOESM2_ESM.tif]
