# Supplementary material for: Embodied mental rotation ability in open- and closed-skill sports: pilot study with a new virtual paradigm
Source: Exp Brain Res. 2024 Jan 20;242(3):653–64. doi: 10.1007/s00221-023-06753-z (PMC10894766; doi:10.1007/s00221-023-06753-z)
Supplement: Supplementary file 3 — Supplementary file3 (DOCX 90 KB) [file 221_2023_6753_MOESM3_ESM.docx]

Embodied mental rotation ability in open- and closed-skill sports:
Pilot study with a new virtual paradigm

*Journal’s name: Experimental Brain Research*

Mai Geisen^1^, Markus Raab^2^, Petra Jansen^3^ and Stefanie Klatt^1^

^1^Institute of Exercise Training and Sport Informatics, German Sport University Cologne, Cologne, Germany

^2^Institute of Psychology, German Sport University Cologne, Cologne, Germany

^3^Institute of Sport Science, University of Regensburg, Regensburg, Germany

Corresponding author: Mai Geisen

E-Mail: [m.geisen@dshs-koeln.de](mailto:m.geisen@dshs-koeln.de)

# Supplementary material

*Graphical presentation of the normal distribution of the results*


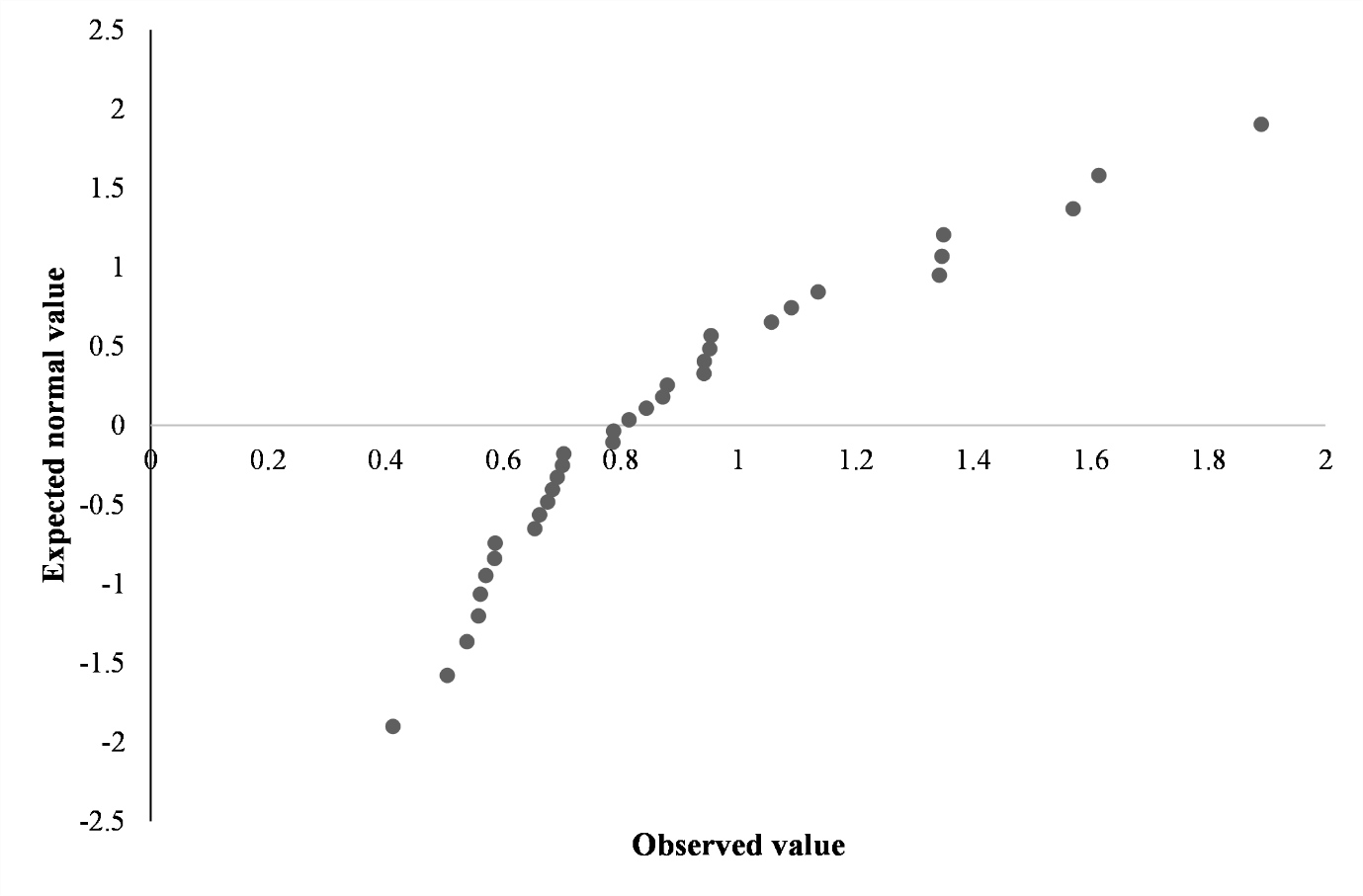


**Fig. 1. Quantile-quantile (Q-Q) plot on reaction time.** The plot shows the expected normal values and observed values on reaction time of participants during the embodied mental rotation task.


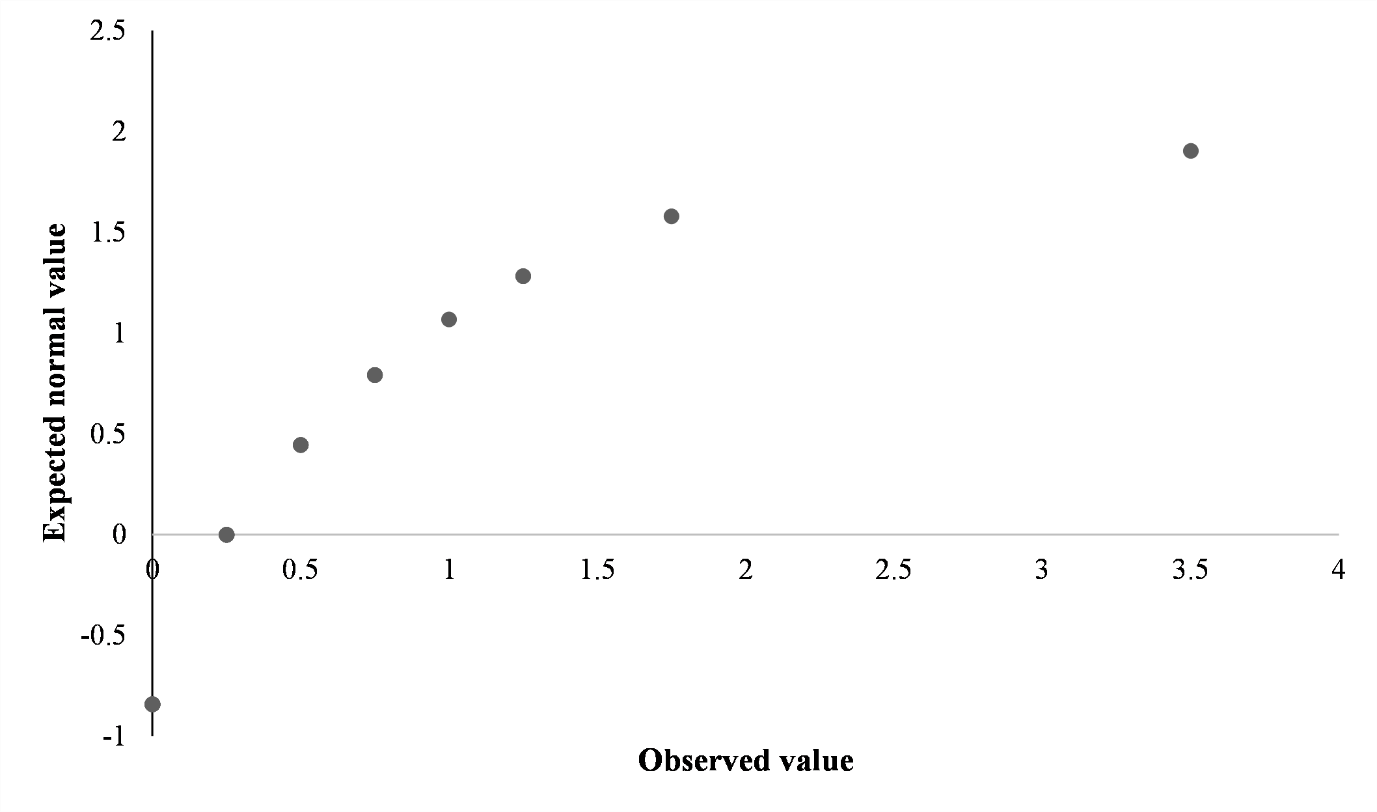


**Fig. 2. Quantile-quantile (Q-Q) plot on number of errors.** The plot shows the expected normal values and observed values on number of errors of participants during the embodied mental rotation task.
